# Prevalence of depression and anxiety and correlations between depression, anxiety, family functioning, social support and coping styles among Chinese medical students

**DOI:** 10.1186/s40359-020-00402-8

**Published:** 2020-04-22

**Authors:** Ruyue Shao, Ping He, Bin Ling, Li Tan, Lu Xu, Yanhua Hou, Liangsheng Kong, Yongqiang Yang

**Affiliations:** 1 School of Clinical Medicine, Chongqing Medical and Pharmaceutical College, No. 82, Daxuecheng Rd, Shapingba Dist, Chongqing, 401331 China; 2 Chongqing Engineering Research Center of Pharmaceutical Sciences, Chongqing, 401331 China; 3 Department of Pharmacology, Chongqing Medical and Pharmaceutical College, No. 82, Daxuecheng Rd, Shapingba Dist, Chongqing, 401331 China; 4 School of Basic Medical Sciences, Chongqing Medical and Pharmaceutical College, No. 82, Daxuecheng Rd, Shapingba Dist, Chongqing, 401331 China; 5 Key Laboratory of Clinical Laboratory Diagnostics (Ministry of Education), College of Laboratory Medicin, Chongqing Medical University, No.1 Yixueyuan Road, Yuzhong District, Chongqing, 400016 China; 6 School of Basic Medical Sciences, Chongqing Medical University, No.1 Yixueyuan Road, Yuzhong District, Chongqing, 400016 China

**Keywords:** Depression, Anxiety, Family function, Social support, Coping style, Medical students

## Abstract

**Background:**

Medical students experience depression and anxiety at a higher rate than the general population or students from other specialties. While there is a growing literature on the high prevalence of depression and anxiety symptoms and about potential risk factors to the prevalence of depression and anxiety symptoms among medical students, there is a paucity of evidence focused on the prevalence of depression and anxiety symptoms and associations with family function, social support and coping styles in Chinese vocational medicine students. This study aims to investigate the prevalence of depression and anxiety symptoms among Chinese medical students and assess the correlation between depression/anxiety symptoms and family function, social support and coping styles.

**Methods:**

A sample of 2057 medical students from Chongqing Medical and Pharmaceutical College in China was investigated with a self-report questionnaire, which included demographic information, Zung self-rating depression scale, Zung Self-Rating Anxiety Scale, Family APGAR Index, Social Support Rating Scale and Trait Coping Style Questionnaire.

**Results:**

The prevalence of depression and anxiety symptoms among the medical students was 57.5 and 30.8%, respectively. Older students(≥20 years) experienced higher levels of depression and anxiety. More depression and anxiety symptoms were exhibited among students with big financial burden, big study-induced stress and poor sleep quality. Students with large employment pressure showed more anxiety symptoms. Students who live alone or had bad relationship with their lovers or classmates or friends showed higher depression and anxiety scores. Depression and anxiety symptoms had highly significant correlations with family functioning, social support and coping style.

**Conclusions:**

Academic staffs should take measures to reduce depression and anxiety among medical students and to provide educational counseling and psychological support for students to cope with these problems.

## Background

Depression is shown to be one of the most common health problems among university students [1, 2]. Medical students experience depression at a higher rate than the general population [3] or students from other specialties [4]. Besides depression, many studies have reported high prevalence of anxiety symptoms among medical students [5–8]. About 30% of medical students suffer from anxiety or depression in Europe [9, 10]. Brazilian studies reported a similar rate, in which 20 to 50% of medical students were found to present with mental disorders [11].

Undoubtedly, medical training is a stressful process which may contribute to the emergence of depression and anxiety [12, 13]. Academic pressure, workload, financial concerns, sleep deprivation, as well as factors interfering in everyday personal life are stressors factors [3, 14]. According to a qualitative-quantitative study conducted at a medical college from August 2016 to March 2017, academic pressure was the major concerns identified by the students when asked about the reasons for psychological distress [15]. Almost all participants mentioned that the huge amount of information and high requirements of medical courses is one of the main causes of high mental distress. At the same time, they found that time for sleep and other social activities was very limited, which only further increased the level of distress [15]. Rosenthal et al. also reported that sleep deprivation may expose students to mood disorders [16]. Medical students also emphasized that finance is an important area to concern. Compared with students belonging to higher sociodemographic backgrounds, psychological distress was higher in which belonging to lower and middle sociodemographic backgrounds [15]. Hojat et al. reported that 42% of first-year and second-year students at Jefferson Medical College have experienced financial hardships in the past 12 months and consider them to be stressful events in their lives [17]. Wege et al. also reported the association between financial problems with psychosomatic symptoms and poor mental health [18]. Depression and anxiety symptoms can adversely influence medical students, including poor academic performance, school dropout, alcohol and substance abuse, internet addiction and suicidal ideation and attempts [19–23].

At the same time, stress during medical training drives medical students to develop certain skills, resources and strategies to cope with these situations, a phenomenon known as coping [24]. Coping refers to the individual cognitive and behavioral strategies to master, reduce or tolerate the internal and external demands of stressful situations [25]. These coping strategies may be positive or negative [26]. Positive coping is an active coping style that focuses on taking constructive actions and changing the stressful situation, and it is typically associated with problem-solving behavior and effective emotion regulation [27]. In contrast, negative coping is a passive style centered on negative appraisals and emotional expression, escape of stressful situations and social isolation [27]. Studies showed that medical students employ both coping styles [28].

While there is a growing literature on the high prevalence of depression and anxiety symptoms and about potential risk factors to the prevalence of depression and anxiety symptoms among medical students, there is a paucity of evidence focused on the prevalence of depression and anxiety symptoms and associations with family function, social support and coping styles in Chinese vocational medicine students. Additionally, in the Chinese context, the medical education system and medical working environment are somewhat different from those in Western or other Asian countries [29]. Excessive number of patients and relatively insufficient number of doctors has resulted in high workload for Chinese doctors. Increasing tension between doctors and patients in recent years frequently lead to violent attacks against medical professionals and a lack of respect from society. These factors could be the cause of worries and mental disorders in Chinese medical students [29, 30]. Thus, the aim of this study was to investigate the prevalence of depression and anxiety symptoms among Chinese medical students and assess the correlation between depression/anxiety symptoms and family function, social support and coping styles among Chinese medical student.

## Methods

### Design and participants

This is a cross-sectional, questionnaire-based descriptive study that was conducted during the year 2018. All medical students from Chongqing Medical and Pharmaceutical College in China were eligible to participate in the study. There were no exclusion criteria. The study was approved by the Ethical Committee of Chongqing Medical and Pharmaceutical College and written informed consent was required from all participants. Participation was voluntary and students were informed about the purpose of the study. Confidentiality was assured and questionnaires were submitted anonymously.

The medical education in mainland China is different from western countries. In China, most undergraduate students are enrolled in medical colleges for a 5-year or 3-year period following high school. 3-year medical education mainly trains doctors at the grassroots level. Chongqing Medical and Pharmaceutical College is a such 3-year medical education college, which enrolls about 3000 students each year, with a total of about 9000 students.

### Instruments

The self-report questionnaire used in this study consisted of six sections or measures, namely demographic information, Zung self-rating depression scale (Zung SDS), Zung Self-Rating Anxiety Scale (Zung SAS), Family APGAR Index (APGAR), Social Support Rating Scale (SSRS) and Trait Coping Style Questionnaire (TCSQ).

#### Demographic information

The demographic section was designed by the research team to collect the general characteristics of medical students, including gender, age, grade, race/ethnicity, place of residence, housing, whether the only child in the family, family characteristics, household income per month, educational levels and occupations of parents, health condition of parents, parents’ way of raising, parents’ care, financial burden during the study, expectations of parents or family members, physical exercise, appetite status, sleep quality, study-induced stress, employment pressure, self-conceived character, having chronic disease or not, satisfaction of specialty and relationship with lovers and classmates or friends.

#### The Zung self-rating depression scale (Zung SDS)

The SDS is a 20-item scale evaluating mood symptoms in the past 7 days. Each item is scored on a Likert scale ranging from 1 to 4 according to the frequency of symptoms over the past week. The score from each item is calculated to obtain the raw score, and the standard score is equal to the raw score multiplied by 1.25. Standard Score is classified as: less than 50, no depression; 50–59, minimal to mild depression; 60–69, moderate to marked depression, greater than 70, severe depression [31–33]. A Chinese version of the SDS was administered in the survey. The reliability and validity of the Chinese version of SDS has been confirmed in previous studies [34, 35].

#### The Zung self-rating anxiety scale (Zung SAS)

The anxiety symptoms among medical students were measured with the SAS, which developed by Zung in 1971 [36]. The SAS questionnaire has 20 self-report questions which were scored on a 4-point Likert scale according to the frequency of symptoms in the past 7 days, ranging from 1 to 4. The score from each item is calculated to obtain the raw score, and the standard score is equal to the raw score multiplied by 1.25. The cut-offs for the SAS standard scores were defined as: less than 50, no anxiety; 50–59, minimal to mild anxiety; 60–69, moderate to marked anxiety, greater than 70, severe anxiety [31, 37]. The Chinese version of the questionnaire has been widely used and demonstrates adequate reliability and validity [5, 38–40].

#### Family APGAR index (APGAR)

The family APGAR index (APGAR) was developed by Smilkstein [41] and has well established reliability and validity [42]. This scale evaluates a family member’s perception of family functioning by assessing his/her satisfaction with family relationships. It includes five parameters: adaptation, partnership, growth, affection and resolve. Three possible answers are allowed(“almost always”, “sometimes”, “hardly ever”), and the score ranges from 0 to 2 points. The points from each item is calculated to obtain the total score. Higher scores indicate better family functioning. A total score of 0–3 suggests severe family dysfunction, 4–6 moderate family dysfunction and 7–10 good family functioning.

#### Social support rating scale (SSRS)

The SSRS was originally developed by Xiao Shuiyuan in 1986 for the Chinese population [43]. It has already been widely used in various studies in different Chinese communities and shown to have good validity and reliability [44–46]. It includes 10 items and evaluates social support in the following three dimensions: Objective support, subjective support, and support utilization. Objective support reflects objective, visible or practical support received in the past. Subjective support reflects the individual emotional experience of being respected, supported and understood in the community. Support utilization reflects the pattern of behavior that an individual uses when seeking social support [46]. Items were mostly rated by 4-point Likert scales. Item scores were added up, generating a final score ranging from 12 to 66. Higher scores indicate stronger social support.

#### Trait coping style questionnaire (TCSQ)

The Trait Coping Style Questionnaire (TCSQ) was used to measure coping strategies, including two domains: positive coping (PC) and negative coping (NC). Each domain consists of 10 items. Each item is ranked on a 5-point Likert-type scale, ranging from 1(absolutely no) to 5(absolutely yes). The higher the one-dimensional scores, the more positive or negative the coping styles are. The TCSQ was developed among the Chinese population in mainland China and has obtained adequate reliability and validity [47].

### Statistical analysis

All analyses were performed using SPSS 21.0 statistical software package (SPSS Inc., Chicago, Illinois). All statistical tests were two-sided((*p*<0.05). All demographic data were analyzed and presented as number(N) and percentage (%). Using Mann-Whitney U-test and Kruskal-Wallis test as appropriate, we compared depression and anxiety severity by demographic variables. Spearman rank order correlation was used to examine correlations among depression, anxiety, family function, social support and coping styles.

To explore the independent effect of different variables on depression and anxiety symptoms, hierarchical regression analysis was used. In model 1, all demographic variables were entered. Dummy variables were set for categorical variables before entering the model. In model 2 to 4, anxiety or depression symptoms, family function, social support were sequentially entered. The results of bivariate correlations showed that the positive coping and negative coping were significantly correlated(r = − 0.129, *p*<0.01). To avoid problems of multicollinearity, positive coping and negative coping were entered respectively in model 5. Standardized estimate(β), F, R^2^ and R^2^-changes (ΔR^2^) for each model were presented.

## Results

Among the 2057 medical students who participated in this research, 603(29.3%) were males, while 1454(70.7%) were females. Their age ranged from 17 to 25(M = 19.76, SD = 1.17). Demographic characteristics of participants are shown in Table 1. The prevalence of depression and anxiety symptoms among the medical students in the present study was 57.5%(SDS index score ≥ 50) and 30.8%(SAS index score ≥ 50), respectively. The mean scores of the SDS and SAS indexes were 51.9 ± 10.1 points and 46.9 ± 7.7 points, respectively. For depression status, the prevalence of each category was 42.5% (no depression), 34.7% (minimal to mild depression), 18% (moderate to marked depression) and 4.9% (severe depression). For anxiety status, the prevalence of each category was 69.2% (no anxiety), 23.9% (minimal to mild anxiety), 6% (moderate to marked anxiety) and 0.8% (severe anxiety).

**Table 1:** Demographic characteristics of the study population

Variables	N(%)
**Gender**	
Male	603(29.3)
Female	1454(70.7)
**Age**	
17–20	835(40.6)
21–25	1222(59.4)
**Grade**	
1st	564(27.4)
2nd	577(28.1)
3rd	916(44.5)
**Race/Ethnicity**	
Han nationality	1926(93.6)
Minority ethic group	131(6.4)
**Place of Residence**	
Urban	922(44.8)
Rural	1135(55.2)
**Housing**	
Alone	134(6.5)
In student residence facility	1201(58.4)
with friends	207(10.1)
With family	515(25)
**Only child in the family**	
Yes	715(34.8)
No	1342(65.2)
**Family characteristics**	
Core family (families consisting of parents and children)	1104(53.7)
Multi-generational family	690(33.5)
Single parent family	175(8.5)
Remarried family	88(4.3)
**Household income per month**	
<RMB 5000	1241(60.3)
RMB 5000–10,000	652(31.7)
>RMB 10000	164(8)
**Paternal education**	
Illiteracy	204(9.9)
Primary school	642(31.2)
Secondary school	1080(52.5)
College and above	129(6.3)
**Maternal education**	
Illiteracy	266(12.9)
Primary school	787(38.3)
Secondary school	910(44.2)
College and above	94(4.6)
**Paternal occupation**^a^	
Manual workers	1688(82.1)
Mental workers	367(17.8)
**Maternal occupation**^a^	
Manual workers	1557(75.7)
Mental workers	500(24.3)
**Paternal health condition**	
Well	690(33.5)
Fare	1092(53.1)
Poor	275(13.4)
**Maternal health condition**	
Well	653(31.7)
Fare	1037(50.4)
Poor	367(17.8)
**Parents’ way of raising you**	
Democracy	1244(60.5)
Authoritarian	372(18.1)
Laissez-faire	441(21.4)
**Parents’ care about you**	
Care	1473(71.6)
General	489(23.8)
Don’t care	95(4.6)
**Financial burden during the study**	
Big	658(32)
Genaral	1109(53.9)
Small	290(14.1)
**Expectations of parents (or family members)**	
Big	1209(58.8)
Genaral	762(37)
Small	86(4.2)
**Physical exercise**	
Never or rarely	575(28)
Occasional	1198(58.2)
Regular (at least 3 times a week, not less than 30 min each time)	284(13.8)
**Appetite status**	
Well	1118(84.4)
Fare	864(42)
Poor	75(3.6)
**Sleep quality**	
Well	880(42.8)
Fare	999(48.6)
Poor	178(8.7)
**Study-induced stress**	
Large	571(27.8)
General	1285(62.5)
Small	200(9.7)
**Employment pressure**	
Large	976(47.4)
General	918(44.6)
Small	163(7.9)
**Self-conceived character**	
Introvert	549(26.7)
Neutral	1070(52)
Extrovert	437(21.2)
**Having chronic disease**	
No	1954(95)
Yes	101(4.9)
**Satisfaction of specialty**	
Satisfied	809(39.3)
General	1123(54.6)
Dissatisfied	125(6.1)
**Relationship with lovers**	
Harmony	582(28.3)
General	333(16.2)
Bad	50(2.4)
Not in love	1092(53.1)
**Relationship with classmates or friends**	
Harmony	1350(65.6)
General	669(32.5)
Bad	38(1.8)

^a^ Occupation: manual workers: workers/farmers/unemployment/commercial service providers/individual businesses/soldier; mental workers: teachers/medical staff/cadres/S&T workers

Comparisons within the various demographic characteristics demonstrated a few significant differences between groups on SDS and SAS scores (Table 2). Older students were more likely to report depression or anxiety symptoms compared to the young students (*p* = 0.002; *p* = 0.001). Depression and anxiety levels showed a non-significant difference by sex. First and second grade students less frequently reported depression or anxiety than did third grade students(*p*<0.001). There was no difference in depression levels between different places of residence; however, students living in rural area were more likely to report anxiety compared to the students living in urban area(*p* = 0.001). Living alone was associated with more depression(*p* = 0.01) and anxiety(*p*<0.001). Although no significant differences were found among paternal or maternal education with respect to symptoms of depression((*p* = 0.258; *p* = 0.726), students whose paternal and maternal educations were low did indicate greater symptoms of anxiety as compared to those whose paternal and maternal educations were high(*p*<0.001; *p* = 0.003). Students who reported that their parents were more authoritarian reported more depression and anxiety symptoms than those who reported that their parents were democracy or laissez-faire(*p*<0.001). Students who reported that their parents cared about them and have big expectations on them were less prone to have depression and anxiety symptoms(*p*<0.05). Students with significant financial burden and study-induced stress exhibited more depression and anxiety symptoms(*p*<0.05). Students with large employment pressure were more likely than those with small employment pressure to report anxiety symptoms(*p*<0.001). Students had poor appetite and sleep reported more depression and anxiety symptoms than those had good or fair appetite and sleep. Students with introvert character reported more depression and anxiety symptoms (*p*<0.001). Bad relationship with lovers or classmates or friends was associated with more depression(*p*<0.05) and anxiety(*p*<0.001). The frequency with which students had bad relationship with lovers was nearly 7 times greater in students with severe anxiety (3 of 17, 17.6%) than in students with minimal to mild anxiety (13 of 492, 2.6%).

**Table 2:** Depression and anxiety scores by respondent characteristics

	Depression classification					Anxiety classification				
Variable	None (Range,<50)(***n*** = 874)	minimal to mild (Range,50–59)(***n*** = 713)	moderate to marked (Range, 60–69)(***n*** = 370)	Severe (Range,≥70) (***n*** = 100)	***P*** Value	None (Range,<50)(***n*** = 1424)	minimal to mild (Range,50–59)(***n*** = 492)	moderate to marked (Range, 60–69)(***n*** = 124)	Severe (Range,≥70) (***n*** = 17)	P Value
**Gender**					0.348					0.693
Male	264(43.8)	210(34.8)	99(16.4)	30(5)		423(70.1)	135(22.4)	36(6)	9(1.5)	
Female	610(42)	503(34.6)	271(18.6)	70(4.8)		1001(68.8)	357(24.6)	88(6.1)	8(0.6)	
**Age**					0.002					0.001
17–20	373(44.7)	311(37.2)	117(14)	34(4.1)		609(72.9)	192(23)	30(3.6)	4(0.5)	
21–25	501(41)	402(32.9)	253(20.7)	66(5.4)		815(66.7)	300(24.5)	94(7.7)	13(1.1)	
**Grade**					<0.001					<0.001
1st	262(46.5)	212(37.6)	72(12.8)	18(3.2)		409(72.5)	131(23.2)	21(3.7)	3(0.5)	
2nd	248(43)	203(35.2)	103(17.9)	23(4)		434(75.2)	120(20.8)	22(3.8)	1(0.2)	
3rd	364(39.7)	298(32.5)	195(21.3)	59(6.4)		581(63.4)	241(26.3)	81(8.8)	13(1.4)	
**Race/Ethnicity**					0.587					0.36
Han nationality	819(42.5)	671(34.8)	345(17.9)	91(4.7)		1338(69.5)	457(23.7)	116(6)	15(0.8)	
Minority ethic group	55(42)	42(32.1)	25(19.1)	9(639)		86(65.6)	35(26.7)	8(6.1)	2(1.5)	
**Place of Residence**					0.607					0.001
Urban	402(43.6)	305(33.1)	173(18.8)	42(4.6)		673(73)	196(21.3)	48(5.2)	5(0.5)	
Rural	472(41.6)	408(35.9)	197(17.4)	58(5.1)		751(66.2)	296(26.1)	76(6.7)	12(1.1)	
**Housing**					0.01					<0.001
Alone	41(30.6)	50(37.3)	32(23.9)	11(8.2)		68(50.7)	37(27.6)	25(18.7)	4(3)	
In student residence facility	521(43.4)	406(33.8)	214(17.8)	60(5)		840(69.9)	289(24.1)	64(5.3)	8(0.7)	
with friends	89(43)	67(32.4)	41(19.8)	10(4.8)		145(70)	48(23.2)	13(6.3)	1(0.5)	
With family	223(43.3)	190(36.9)	83(16.1)	19(3.7)		371(72)	118(22.9)	22(4.3)	4(0.8)	
**Only child in the family**					0.938					0.442
Yes	309(43.2)	239(33.4)	127(17.8)	40(5.6)		488(68.3)	174(24.3)	46(6.4)	7(1)	
No	565(42.1)	474(35.3)	243(18.1)	60(4.5)		936(69.7)	318(23.7)	78(5.8)	10(0.7)	
**Family characteristics**					0.757					0.462
Core family (families consisting of parents and children)	465(42.1)	375(34)	209(18.9)	55(5)		773(70)	255(23.1)	68(6.2)	8(0.7)	
Multi-generational family	297(43)	247(35.87)	115(16.7)	31(4.5)		466(67.5)	181(26.2)	37(5.4)	6(0.9)	
Single parent family	78(44.6)	57(32.6)	31(17.7)	9(5.1)		119(68)	39(22.3)	14(8)	3(1.7)	
Remarried family	34(38.6)	34(38.6)	15(17)	5(5.7)		66(75)	17(19.3)	5(5.7)	0(0)	
**Household income per month**					0.241					0.268
<RMB 5000	510(41.1)	438(35.3)	225(18.1)	68(5.5)		841(67.8)	317(25.54)	72(5.8)	11(0.9)	
RMB 5000–10,000	290(44.5)	220(33.7)	115(17.5)	27(4.1)		467(71.6)	142(21.8)	39(6)	4(0.6)	
>RMB 10000	74(45.1)	55(33.5)	30(18.3)	5(3)		116(70.7)	33(20.1)	13(7.9)	2(1.2)	
**Paternal education**					0.258					<0.001
Illiteracy	79(38.7)	75(36.8)	33(16.2)	17(8.3)		109(53.4)	74(36.3)	17(8.3)	4(2)	
Primary school	265(41.2)	222(34.5)	123(19.1)	33(5.1)		435(67.7)	161(25)	41(6.4)	6(0.9)	
Secondary school	468(43.3)	377(34.9)	190(17.6)	46(4.3)		787(72.8)	228(21.1)	59(5.5)	7(0.6)	
College and above	62(48.1)	39(30.2)	24(18.6)	4(3.1)		93(72.1)	29(22.5)	7(5.4)	0(0)	
**Maternal education**					0.726					0.003
Illiteracy	117(44)	96(36.1)	36(13.5)	17(6.4)		165(62)	78(29.3)	20(7.5)	3(1.1)	
Primary school	331(42.1)	262(33.3)	151(19.2)	43(5.5)		544(69.1)	189(24)	47(6)	7(0.9)	
Secondary school	387(42.5)	324(35.6)	162(17.8)	37(4.1)		657(72.2)	201(22.1)	47(5.2)	5(0.5)	
College and above	39(41.5)	31(33)	21(22.3)	3(3.2)		58(61.7)	24(25.5)	10(10.6)	2(2.1)	
Paternal occupation*					0.679					0.692
Manual workers	718(42.5)	578(34.2)	309(18.3)	84(5)		1163(68.9)	418(24.7)	98(5.8)	10(0.6)	
Mental workers	156(42.4)	135(36.7)	61(16.6)	16(4.3)		261(70.9)	74(20.1)	26(7.1)	7(1.9)	
Maternal occupation*					0.863					0.977
Manual workers	657(42.2)	545(35)	285(18.3)	70(4.5)		1074(69)	387(24.9)	85(5.5)	11(0.7)	
Mental workers	217(43.4)	168(33.6)	85(17)	30(6)		350(70)	105(21)	39(7.8)	6(1.2)	
**Paternal health condition**					0.062					0.052
Well	315(45.7)	225(32.6)	118(17.1)	32(4.6)		501(72.6)	141(20.4)	39(5.7)	9(1.3)	
Fair	456(41.8)	389(35.6)	1937.7)	54(4.9)		744(68.1)	280(25.6)	62(5.7)	6(0.5)	
Poor	103(37.5)	99(36)	59(21.5)	14(5.1)		179(65.1)	71(25.8)	23(8.4)	2(0.7)	
**Maternal health condition**					0.567					0.222
Well	292(44.7)	208(31.9)	119(18.2)	34(5.2)		469(71.8)	137(21)	38(5.8)	9(1.4)	
Fair	431(41.6)	382(36.8)	177(17.1)	47(4.5)		712(68.7)	257(24.8)	62(6)	6(0.6)	
Poor	151(41.1)	123(33.5)	74(20.2)	19(5.2)		243(66.2)	98(26.7)	24(6.5)	2(0.5)	
**Parents’ way of raising you**					<0.001					<0.001
Democracy	570(45.8)	438(35.2)	182(14.6)	54(4.3)		928(74.6)	255(20.5)	52(4.2)	9(0.7)	
Authoritarian	118(31.7)	127(34.1)	101(27.2)	26(7)		202(54.3)	121(32.5)	44(11.8)	5(1.3)	
Laissez-faire	186(42.2)	148(33.6)	87(19.7)	20(4.5)		294(66.7)	116(26.3)	28(6.3)	3(0.7)	
**Parents care about you**					<0.001					<0.001
Care	686(46.6)	490(33.3)	241(16.4)	56(3.8)		1098(74.5)	309(21)	56(3.8)	10(0.7)	
General	161(32.9)	188(38.4)	107(21.9)	33(6.7)		290(59.3)	147(30.1)	47(9.6)	5(1)	
Don’t care	27(28.4)	35(36.8)	22(23.2)	11(11.6)		36(37.9)	36(37.9)	21(22.1)	2(2.1)	
**Financial burden during the study**					0.002					<0.001
Big	256(38.9)	220(33.4)	142(21.6)	40(6.1)		413(62.8)	186(28.3)	51(7.8)	8(1.2)	
General	478(43.1)	396(35.7)	190(17.1)	45(4.1)		776(70)	263(23.7)	61(5.5)	9(0.8)	
Small	140(48.3)	97(33.4)	38(13.1)	15(5.2)		235(81)	43(14.8)	12(4.1)	0(0)	
**Expectations of parents (or family members)**					0.035					<0.001
Big	519(42.9)	424(35.1)	212(17.5)	54(4.5)		860(71.1)	280(23.2)	60(5)	9(0.7)	
General	326(42.8)	263(34.5)	135(17.7)	38(5)		518(68)	191(25.1)	47(6.2)	6(0.8)	
Small	29(33.7)	26(30.2)	23(26.7)	8(9.3)		46(53.5)	21(24.4)	17(19.8)	2(2.3)	
**Physical exercise**					0.246					0.055
Never or rarely	223(38.8)	218(37.9)	104(18.1)	30(5.2)		377(65.6)	152(26.4)	38(6.6)	8(1.4)	
Occasional	529(44.2)	391(32.6)	220(18.4)	58(4.8)		841(70.2)	280(23.4)	68(5.7)	9(0.8)	
Regular (at least 3 times a week, not less than 30 min each time)	122(43)	104(36.6)	46(16.2)	12(4.2)		206(72.5)	60(21.1)	18(6.3)	0(0)	
**Appetite status**					<0.001					<0.001
Well	516(46.2)	372(33.3)	192(17.2)	38(3.4)		845(75.6)	216(19.3)	48(4.3)	9(0.8)	
Fair	337(39)	316(36.6)	161(18.6)	50(5.8)		557(64.5)	246(28.5)	55(6.4)	6(0.7)	
Poor	21(28)	25(33.3)	17(22.7)	12(16)		22(29.3)	30(40)	21(28)	2(2.7)	
**Sleep quality**					0.004					<0.001
Well	400(45.5)	297(33.8)	145(16.5)	38(4.3)		676(76.8)	149(16.9)	46(5.2)	9(1)	
Fair	415(41.5)	348(34.8)	187(18.7)	49(4.9)		668(66.9)	271(27.1)	55(5.5)	5(0.5)	
Poor	59(33.1)	68(38.2)	38(21.3)	13(7.3)		80(44.9)	72(40.4)	23(12.9)	3(1.7)	
**Study-induced stress**					<0.001					<0.001
Large	197(34.5)	211(37)	120(21)	43(7.5)		325(56.9)	182(31.9)	54(9.5)	10(1.8)	
General	583(45.3)	434(33.7)	218(17)	51(4)		944(73.4)	278(21.6)	57(4.4)	7(0.5)	
Small	94(47)	68(34)	32(16)	6(3)		155(77.5)	32(16)	13(6.5)	0(0)	
**Employment pressure**					0.059					<0.001
Large	390(40)	348(35.7)	185(19)	53(5.4)		624(63.9)	275(28.2)	68(7)	9(0.9)	
General	415(45.2)	306(33.3)	160(17.4)	37(4)		676(73.6)	188(20.5)	46(5)	8(0.9)	
Small	69(42.3)	59(36.2)	25(15.3)	10(6.1)		124(76.1)	29(17.8)	10(6.1)	0(0)	
**Self-conceived character**					<0.001					<0.001
Introvert	190(34.5)	202(36.7)	118(21.5)	40(7.3)		330(60)	156(28.4)	53(9.6)	11(2)	
Neutral	464(43.4)	377(35.2)	190(17.8)	39(3.6)		768(71.8)	252(23.6)	45(4.2)	5(0.5)	
Extrovert	220(50.3)	134(30.7)	62(14.2)	21(4.8)		326(74.6)	84(19.2)	26(5.9)	1(0.2)	
**Having chronic disease**					0.34					0.12
No	840(42.9)	666(34)	354(18.1)	96(4.9)		1362(69.6)	459(23.5)	119(6.1)	16(0.8)	
Yes	34(33.7)	47(46.5)	16(15.8)	4(4)		62(61.4)	33(32.7)	5(5)	1(1)	
**Satisfaction of specialty**					0.082					0.116
Satisfied	371(45.9)	266(32.9)	128(15.8)	44(5.4)		583(72.1)	165(20.4)	52(6.4)	9(1.1)	
General	454(40.4)	398(35.4)	220(19.6)	51(4.5)		761(67.8)	291(25.9)	64(5.7)	7(0.6)	
Dissatisfied	49(39.2)	49(39.2)	22(17.6)	5(4)		80(64)	36(28.8)	8(6.4)	1(0.8)	
**Relationship with lovers**					<0.001					<0.001
Harmony	266(45.7)	179(30.8)	108(18.6)	29(5)		401(68.9)	137(23.5)	38(6.5)	6(1)	
General	111(33.3)	122(36.6)	72(21.6)	28(8.4)		182(54.7)	103(30.9)	42(12.6)	6(1.8)	
Bad	19(38)	16(32)	9(18)	6(12)		25(50)	13(26)	9(18)	3(6)	
Not in love	478(43.8)	396(36.3)	181(16.6)	37(3.4)		816(74.7)	239(21.9)	35(3.2)	2(0.2)	
**Relationship with classmates or friends**					0.001					<0.001
Harmony	594(44)	482(35.7)	219(16.2)	55(4.1)		1014(75.1)	268(19.9)	59(4.4)	9(0.7)	
General	271(40.5)	214(32)	146(21.8)	38(5.7)		401(59.9)	211(31.5)	52(7.8)	5(0.7)	
Bad	9(23.7)	17(44.7)	5(13.2)	7(18.4)		9(23.7)	13(34.2)	13(34.2)	3(7.9)	

Bivariate correlations demonstrated in Table 3 suggested that depression and anxiety symptoms had highly significant correlations with family functioning, social support and coping style. Depression and anxiety were significantly positively correlated with each other(r = 0.403, *p*<0.01). Depression significantly negatively correlated with family functioning and all five dimensions (i.e., adaptation, partnership, growth, affection, resolve) (r ranged − 0.117 to − 0.031, *p*<0.05) and social support and all its three dimensions (i.e., objective support, subjective support, support utilization) (r ranged − 0.089 to − 0.037, *p*<0.01). Additionally, depression significantly negatively correlated with positive coping(r = − 0.102, *p*<0.01) and significantly positively correlated with negative coping(r = 0.167, *p*<0.01). Similarly, anxiety significantly negatively correlated with family functioning and all five dimensions(i.e., adaptation, partnership, growth, affection, resolve) (r ranged − 0.264 to − 0.122, *p*<0.01) and social support and all its three dimensions(i.e., objective support, subjective support, support utilization) (r ranged − 0.17 to − 0.102, *p*<0.01). Also, anxiety significantly negatively correlated with positive coping(r = − 0.308, *p*<0.01) and significantly positively correlated with negative coping(r = 0.245, *p*<0.01).

**Table 3:** Correlation matrix of depression, anxiety, family functioning, social support and trait coping style

Variable	1	2	3	4	5	6	7	8	9	10	11	12	13	14
1.Zung SDS	1													
2.Zung SAS	0.403^**^	1												
3.Adaptation	−0.031^**^	−0.122^**^	1											
4.Partnership	− 0.050^*^	− 0.139^**^	0.526^**^	1										
5.Growth	−0.083^**^	− 0.208^**^	0.326^**^	0.354^**^	1									
6.Affection	− 0.089^**^	− 0.191^**^	0.384^**^	0.441^**^	0.418^**^	1								
7.Resolve	−0.113^**^	−0.260^**^	0.314^**^	0.400^**^	0.369^**^	0.477^**^	1							
8.APGAR	−0.117^**^	−0.264^**^	0.677^**^	0.750^**^	0.677^**^	0.754^**^	0.708^**^	1						
9.Objective support	−0.037^**^	−0.102^**^	0.116^**^	0.099^**^	0.105^**^	0.129^**^	0.100^**^	0.146^**^	1					
10.Subjective support	−0.081^**^	−0.130^**^	0.110^**^	0.109^**^	0.119^**^	0.157^**^	0.140^**^	0.170^**^	0.288^**^	1				
11.Support utilization	−0.058^**^	−0.152^**^	0.126^**^	0.127^**^	0.142^**^	0.165^**^	0.155^**^	0.197^**^	0.170^**^	0.189^**^	1			
12.SSRS	−0.089^**^	−0.170^**^	0.155^**^	0.148^**^	0.163^**^	0.202^**^	0.177^**^	0.227^**^	0.593^**^	0.889^**^	0.445^**^	1		
13.Positive coping	−0.102^**^	−0.308^**^	0.073^**^	0.106^**^	0.150^**^	0.133^**^	0.186^**^	0.180^**^	0.207^**^	0.176^**^	0.227^**^	0.265^**^	1	
14.Negative coping	0.167^**^	0.245^**^	−0.168^**^	−0.193^**^	−0.146^**^	− 0.181^**^	−0.192^**^	− 0.240^**^	−0.038	− 0.103^**^	−0.177^**^	− 0.132^**^	−0.129^**^	1

Note: *Zung SDS* The Zung self-rating depression scale, *Zung SAS* The Zung Self-Rating Anxiety Scale, *APGAR* Family APGAR Index, *SSRS* Social Support Rating Scale, *TCSQ* Trait Coping Style Questionnaire

^*^p<0.05

^**^p<0.01

The results of the hierarchical regression of depression and anxiety symptoms are presented in Tables 4 and 5. After adjusting demographic variables, negative coping was positively associated with depression(β = 0.226, *p*<0.001). A 0.226-unit rise in depression was associated with each unit increase in negative coping style (Table 4).

**Table 4:** Hierarchical linear regression analysis of independent factors correlated to depression symptoms

Variables	Model 1(β)	Model 2(β)	Model 3(β)	Model 4(β)	
**Model 1**					
Gender:Male (ref:female)	−0.446	− 0.452	− 0.43	− 0.469	− 0.006
Age	0.108	0.12	0.123	0.119	0.129
Grade	0.973^**^	1.02^**^	1.019^**^	1.018^**^	0.98^**^
Race/Ethnicity:Han nationality (ref: Minority ethic group)	0.176	0.15	0.125	0.146	0.068
Place of Residence:Urban (ref:rural)	0.411	0.46	0.429	0.425	0.262
Housing: In student residence facility (ref:alone)	−1.18	−1.2	− 1.182	− 1.159	− 1.263
Housing:with friends (ref:alone)	−2.237^*^	−2.179	− 2.124	−2.087	− 2.164
Housing:with family (ref:alone)	−1.487	− 1.498	− 1.464	−1.448	− 1.572
Only child in the family: Yes (ref:no)	0.064	0.072	0.019	0.028	0.025
Family characteristics: Multi-generational family (ref:core family)	−0.665	− 0.644	− 0.653	− 0.643	− 0.682
Family characteristics: Single parent family (ref:core family)	−1.035	−1.041	− 1.054	− 1.075	− 0.923
Family characteristics: Remarried family (ref:core family)	−0.217	− 0.22	− 0.212	− 0.206	− 0.164
Household income per month: RMB 5000–10,000(ref:<RMB 5000)	− 1.266*	−1.274^*^	− 1.284^*^	−1.299^*^	− 1.232^*^
Household income per month:>RMB 10000(ref:<RMB 5000)	−0.846	− 0.937	− 0.906	− 0.9	− 1.036
Paternal education: Primary school (ref:illiteracy)	−0.204	− 0.242	− 0.281	−0.299	− 0.132
Paternal education: Secondary school (ref:illiteracy)	−0.653	− 0.664	−0.698	− 0.709	−0.382
Paternal education: College and above (ref:illiteracy)	−0.396	−0.342	− 0.367	−0.31	− 0.135
Maternal education: Primary school (ref:illiteracy)	1.509^*^	1.492^*^	1.494^*^	1.485^*^	1.526^*^
Maternal education: Secondary school (ref:illiteracy)	1.097	1.127	1.136	1.123	1.23
Maternal education: College and above (ref:illiteracy)	2.076	2.131	2.158	2.151	1.841
Paternal occupation: Mental workers (ref: Manual workers)	−0.455	−0.49	− 0.494	−0.466	− 0.588
Maternal occupation: Mental workers (ref: Manual workers)	0.672	0.642	0.638	0.654	0.577
Paternal health condition	0.218	0.216	0.224	0.248	0.17
Maternal health condition	−0.348	− 0.378	−0.395	− 0.389	−0.471
Parents’ way of raising you: Authoritarian (ref:democracy)	2.394^**^	2.243^**^	2.212^**^	2.213^**^	2.155^**^
Parents’ way of raising you: Laissez-faire (ref:democracy)	0.18	0.067	0.037	0.064	0.012
Parents care about you	1.498^**^	1.348^**^	1.331^**^	1.34^**^	1.355^**^
Financial burden during the study	−0.368	−0.341	−0.333	−0.322	− 0.378
Expectations of parents (or family members)	0.091	0.074	0.074	0.065	0.099
Physical exercise	0.187	0.216	0.238	0.227	0.379
Appetite status	1.032^*^	1.006^*^	1.003^*^	0.991^*^	0.99*
Sleep quality	0.383	0.365	0.336	0.319	0.309
Study-induced stress	−1.173^**^	−1.146^**^	−1.144^**^	−1.161^**^	−0.887^*^
Employment pressure	−0.093	− 0.063	− 0.055	− 0.045	0.167
Self-conceived character: Neutral (ref:introvert)	−1.678^**^	− 1.613^**^	− 1.603^**^	−1.576^**^	− 1.439^**^
Self-conceived character: Extrovert (ref:introvert)	−1.968^**^	− 1.9^**^	−1.859^**^	− 1.795^**^	−1.725^**^
Having chronic disease: Yes (ref:no)	1.153	1.158	1.181	1.18	1.074
Satisfaction of specialty	0.32	0.263	0.241	0.243	0.187
Relationship with lovers	0.299	0.297	0.334	0.305	0.382^*^
Relationship with classmates or friends	0.754	0.656	0.614	0.566	0.564
**Model 2**					
Family function		−0.201	−0.186	−0.179	−0.049
**Model 3**					
Social support			−0.034	− 0.026	− 0.028
**Model 4**					
Positive coping				−0.033	
Negative coping					0.226^**^
**F**	4.123^**^	4.116^**^	4.042^**^	3.973^**^	5.064^**^
**R2**	0.076	0.077	0.078	0.078	0.098
**ΔR2**	0.076	0.002	0	0	0.02

^*^p<0.05

^**^p<0.01

**Table 5:** Hierarchical linear regression analysis of independent factors correlated to anxiety symptoms

Variables	Model 1(β)	Model 2(β)	Model 3(β)	Model 4(β)	
**Model 1**					
Gender:Male (ref:female)	0.008	−0.01	0.02	−0.16	0.459
Age	0.099	0.134	0.139	0.119	0.145
Grade	0.782^**^	0.924^**^	0.923^**^	0.92^**^	0.884^**^
Race/Ethnicity:Han nationality (ref: Minority ethic group)	0.684	0.602	0.569	0.665	0.375
Place of Residence:Urban (ref:rural)	−0.793^*^	−0.644	− 0.687^*^	− 0.705^*^	− 0.855^*^
Housing:In student residence facility (ref:alone)	−2.696^**^	−2.755^**^	−2.73^**^	−2.622^**^	− 2.812^**^
Housing:with friends (ref:alone)	−3.788^**^	−3.612^**^	−3.536^**^	− 3.365^**^	− 3.576^**^
Housing:with family (ref:alone)	−2.376^**^	−2.407^**^	− 2.36^**^	− 2.288^**^	− 2.469^**^
Only child in the family: Yes (ref:no)	0.34	0.364	0.291	0.331	0.247
Family characteristics: Multi-generational family (ref:core family)	0.171	0.235	0.221	0.267	0.193
Family characteristics: Single parent family (ref:core family)	−0.104	−0.122	− 0.141	− 0.238	− 0.009
Family characteristics: Remarried family (ref:core family)	− 1.685^*^	−1.695^*^	− 1.684^*^	− 1.659^*^	−1.635^*^
Household income per month: RMB 5000–10,000(ref:<RMB 5000)	−0.132	− 0.157	− 0.171	−0.239	− 0.118
Household income per month:>RMB 10000(ref:<RMB 5000)	−0.613	− 0.89	−0.848	− 0.819	−0.979
Paternal education: Primary school (ref:illiteracy)	− 0.877	− 0.993	− 1.047	− 1.131^*^	− 0.897
Paternal education: Secondary school (ref:illiteracy)	− 1.391^*^	−1.423^*^	− 1.471^**^	−1.519^**^	− 1.152^*^
Paternal education: College and above (ref:illiteracy)	−1.834^*^	− 1.67	−1.704	− 1.44	−1.47
Maternal education: Primary school (ref:illiteracy)	0.302	0.25	0.253	0.214	0.285
Maternal education: Secondary school (ref:illiteracy)	0.487	0.579	0.591	0.531	0.685
Maternal education: College and above (ref:illiteracy)	2.357^*^	2.525^**^	2.562^**^	2.532^**^	2.243^*^
Paternal occupation: Mental workers (ref: Manual workers)	0.172	0.065	0.06	0.187	0.034
Maternal occupation: Mental workers (ref: Manual workers)	−0.683	− 0.771^*^	−0.778^*^	− 0.7	− 0.839^*^
Paternal health condition	− 0.259	− 0.263	− 0.253	−0.139	− 0.307^**^
Maternal health condition	−0.129	− 0.217	−0.242	− 0.213	−0.318
Parents’ way of raising you: Authoritarian (ref:democracy)	1.884^**^	1.425^**^	1.383^**^	1.385^**^	1.325
Parents’ way of raising you: Laissez-faire (ref:democracy)	0.545	0.199	0.159	0.282	0.109
Parents care about you	1.45^**^	0.995^**^	0.971^**^	1.017^**^	0.996^**^
Financial burden during the study	−0.503^*^	−0.42	− 0.41	−0.357	− 0.455
Expectations of parents (or family members)	0.725^*^	0.672^*^	0.673^*^	0.632^*^	0.698^*^
Physical exercise	0.298	0.387	0.417	0.368	0.559^*^
Appetite status	1.206^**^	1.128^**^	1.124^**^	1.067^**^	1.11^**^
Sleep quality	1.341^**^	1.287^**^	1.247^**^	1.17^**^	1.22^**^
Study-induced stress	−1.395^**^	−1.315^**^	−1.312^**^	− 1.388^**^	− 1.053^**^
Employment pressure	−0.996^**^	−0.903^**^	− 0.892^**^	− 0.848^**^	− 0.668^**^
Self-conceived character: Neutral (ref:introvert)	− 1.908^**^	− 1.713^**^	− 1.698^**^	−1.574^**^	− 1.533^**^
Self-conceived character: Extrovert (ref:introvert)	−1.523^**^	− 1.318^**^	−1.261^**^	− 0.965^*^	− 1.126*
Having chronic disease: Yes (ref:no)	0.892	0.909	0.94	0.936	0.833
Satisfaction of specialty	0.216	0.043	0.012	0.021	−0.042
Relationship with lovers	0.769^**^	0.763^**^	0.815^**^	0.682^**^	0.863^**^
Relationship with classmates or friends	2.138^**^	1.839^**^	1.782^**^	1.557^**^	1.731^**^
**Model 2**					
Family function		−0.609**	−0.589^**^	−0.557	−0.451^**^
**Model 3**					
Social support			−0.047^*^	−0.011	−0.041
**Model 4**					
Positive coping				−0.151^**^	
Negative coping					0.227^**^
**F**	14.935^**^	16.794^**^	16.517^**^	17.662^**^	19.189^**^
**R2**	0.229	0.255	0.256	0.274	0.291
**ΔR2**	0.229	0.026	0.002	0.018	0.035

^*^p<0.05

^**^p<0.01

Table 5 indicates that three independent variable blocks significantly predicted anxiety symptoms: family function(R^2^ change = 0.026, *p*<0.001), social support(R^2^ change = 0.002, *p*<0.05), positive coping(R^2^ change = 0.018, *p*<0.001) and negative coping(R^2^ change = 0.035, *p*<0.001). After adjusting demographic variables, negative coping positively associated with anxiety(β = 0.227, *p*<0.001), while family function, social support and positive coping were negatively correlated to it(β = − 0.609, *p*<0.001, − 0.047, *p*<0.05 and − 0.151, *p*<0.001, respectively).

## Discussion

The results of this study revealed a high prevalence of depression (57.5%) among Chinese medical students from Chongqing Medical and Pharmaceutical College. Several previous studies have demonstrated similar high depression rates among medical students [48–50]. Fawzy et al. [14] reported an even higher rate (65%). On the contrary, many other previous studies demonstrated lower rates of depression ranging from 15 to 24% in USA [51], 30.6% in Cameroon [52], 29.5% in Turkey [53], 37.2% in Malaysia [54]. Also, the prevalence of depression of our sample (57.5%) was much higher than the global prevalence (28.0%) estimated by a meta-analysis of 62,728 medical students and 1845 non-medical students pooled across 77 studies [55] and aggregate prevalence (11.0%) estimated by a meta-analysis of 10,147 medical students in Asia [56]. According to our results, the overall anxiety symptom prevalence in our sample was 30.8%, which was lower than that of another group of Chinese medical students (47.3%) whose anxiety was measured by the same scale [5]. Also, the prevalence of anxiety of our sample (30.8%) was similar to the global prevalence (33.8%) estimated by a meta-analysis of 40,348 medical students across 69 studies [57]. Numerous studies showed that the prevalence of anxiety among medical students was 43.7% in Pakistan [8], 29.4% in Israel [6], 44% in Malaysia [7]. Additionally, we found that depression and anxiety were significantly positively correlated with each other. This finding is supported by other studies which shown that people with high anxiety were more likely to become more easily depressed [58] and major depressive disorder has high comorbidity with numerous anxiety disorders in general population [3].

In this study, we observed that older students(≥20 years) experienced higher levels of depression and anxiety. In accordance, Shamsuddin et al. [54] suggested that students in the older age group (≥20 years) had higher depression and anxiety scores. Bostanci et al. [59] also reported higher scores of depression among senior Turkish students compared to the freshmen. We also reported higher scores of depression and anxiety among students in the third grade compared to those in the first and second grade.

This might suggest a decrease of psychological health in medical students. These findings were similar to the previous studies. Iqbal et al. [48] reported that fifth semester students had higher depression and anxiety scores than second and forth semester students. Baldassin et al. [60] found that Brazilian medical students in the internship period(5th and 6th years) exhibited higher depression levels in comparison to students both in the basic(1st and 2nd years) and intermediate(3rd and 4th years) periods. Chongqing Medical and Pharmaceutical College is a three-year medical education college in China which mainly trains doctors at the grassroots level. Grades 1 and 2 students learn the courses of medicine, and Grade 3 students enter clinical practice. Depression and anxiety may be more common among students in the third grade as a result of excessive workload of both paraclinical and clinical subjects, increasing tension between doctors and patients in recent years in China and worry about not attaining their goal of being a doctor. In contrast, some studies identified that preclinical students exhibited higher levels of depression and anxiety compared to students in higher years of study [14, 61] and some identified no difference in depression and anxiety prevalence according to students’ year of study [3]. These conflicting findings may be due to differences in study populations. Nevertheless, depression and anxiety experienced by medical students could be a predisposing factor to burnout during residency or postgraduate training [62].

In our study, we reported that more depression and anxiety symptoms were exhibited among students with significant financial burden, high level of academic stress and poor sleep quality. Students with large employment pressure showed more anxiety symptoms. Previous studies have suggested that academic pressure, workload, sleep deprivation and financial concerns may have an adverse effect on students’ mental health [3, 14, 63, 64]. Wege et al. [18] indicated that expected financial hardships were significantly associated with mental health disorders and psychosomatic symptoms. Association between worrying about the future with anxiety scores had been reported [65]. Getting into medical school required to the students changing their lifestyle [3]. One of these changes is living away from their families and friends. In this case housing accommodations can impact the medical students’ mental health. Our hypothesis that students who live alone have higher levels of depression and anxiety has been confirmed. Furthermore we confirmed the hypotheses that students had bad relationship with their lovers or classmates or friends showed higher depression and anxiety scores, which was consistent with previous studies [65, 66].

Social support has been defined as “a social network’s provision of psychological and material resources intended to benefit an individual’s ability to cope with stress” by Cohen [67]. The negative relationship between social support and psychological symptoms (depression, anxiety) supports the findings of previous studies [47, 58]. It is generally believed that positive social support is an important aspect of psychological adjustment that could help buffer the pathogenic effects of stress [58, 68]. It was reported the lack of social support associated with reduced positive emotion and experience, and lessened psychological well-being of medical students [65, 69]. Kjeldstadli et al. [70] also indicated that medical students who perceived medical school as interfering less with their social and personal lives were psychologically more stable.

Family is an important source of support for Chinese medical students. Our study found a negative relationship between family function and psychological symptoms (depression, anxiety). This result was aligned with previous studies suggesting an association between family dysfunction and depressive symptoms [71]. Wickrama KA et al. [72] also reported that positive family functioning reduced mothers’ depressive symptoms in Tsunami-affected families. The emotional comforts of the family function may help to improve physical and psychological well-being of medical students.

Coping refers to the individual cognitive and behavioral strategies to master, reduce or tolerate the internal and external demands of stressful situations [25]. The term “coping styles” is applied to more consistent tendencies to cope in a particular way [73]. Coping styles are broadly grouped into positive coping (PC) and negative coping (NC) styles [74]. The results of this study indicated that positive coping was negatively related to psychological symptoms (depression, anxiety), while negative coping was positively related to them. The results of hierarchical regression also revealed that coping styles were the independent predictors of psychological symptoms. These findings were congruent with previous studies in China. Luo et al. reported that negative coping was positively correlated to psychological symptoms and positive coping was negatively correlated to them among Chinese nurse students [47]. However, one study in Brazil reported either PC or NC styles to be correlated to more depressive symptoms [75], and another study in the United States found null effect of coping styles on mental health [76]. These mixed findings may be due to different countries.

There are several limitations in the present study. First, the study design was cross-sectional, which precluding definitive conclusions regarding the direction of causality between social support, family functioning, coping styles and psychological morbidity. Further longitudinal studies are expected to explore the causalities. Second, the study population consisted only of medical students in one Chinese medical school and therefore may not be extended directly to other settings. Medical students from other universities will be investigated in our further research. Third, all questionnaires were self-report and therefore the inherent limitations of self-reported measures should be noted. Fourth, all medical students from Chongqing Medical and Pharmaceutical College in China were eligible to participate in the study and there were no exclusion criteria. This may lead to a self-selection bias, as medical students with depression or anxiety symptoms may be less motivated to completed the questionnaires, or on the other hand, they may be more likely to participate since the topic is relevant to them. Finally, the effect of psychological mechanism on depression and anxiety of Chinese medical students has not been studied in this study. Studies have found that certain psychological mechanisms are related to depression and anxiety. Studies showed that depressive symptoms and rumination in individuals are usually highly correlated. In one study, individuals who had more ruminative responses about the 1989 Loma Prieta Earthquake were more likely to have prolonged high levels of depression than those who did not develop these thought patterns [77]. In another study, rumination was associated with longer and more severe depression after experiencing stress [78]. This aspect can be further studied in our future research.

## Conclusions

The present study reported a relatively high prevalence of depression and anxiety symptoms in a sample of Chinese medical students. Multiple factors were related to depression and anxiety symptoms. Supportive social relationships, positive family function and positive coping style may play an important role in reducing the stresses and improving their mental well-being. These results are important for both students and academic staffs. By broadening social relationships and adopting more positive coping and less negative coping skills, depression and anxiety symptoms may be prevented or at least diminished among medical students. In addition, academic staffs should take measures and interventions to reduce depression and anxiety among medical students and to provide educational counseling and psychological support for students to cope with these problems.

## Data Availability

The detasets used analysed during the current study are available from the corresponding author on reasonable request.

## Abbreviations

SDS: Self-rating depression scale
SAS: `Self-rating anxiety scale
APGAR: adaptation, partnership, growth, affection, resolve
SSRS: Social support rating scale
TCSQ: Trait Coping Style Questionnaire
